# Shining a Light on a Hidden Population: Social Functioning and Mental Health in Women Reporting Autistic Traits But Lacking Diagnosis

**DOI:** 10.1007/s10803-022-05583-2

**Published:** 2022-05-20

**Authors:** Hannah L. Belcher, Sharon Morein-Zamir, Steven D. Stagg, Ruth M. Ford

**Affiliations:** 1 IOPPN, King’s College London, 16 De Crespigny Park, London, SE5 8AB UK; 2 School of Psychology and Sport Science, Anglia Ruskin University, East Road, Cambridge, CB1 1PT UK

**Keywords:** Autism spectrum conditions, Female phenotype of autism, Late diagnosis, Autism masking, Psychiatric comorbidity, Misdiagnosis

## Abstract

Female Phenotype Theory (FPT) suggests that autistic women often present with less obvious social impairments than autistic men. We examined the possibility of an exaggerated female phenotype among undiagnosed but probably autistic women. In two nationwide online surveys, we compared self-reported social functioning and mental health between diagnosed autistic women and women without diagnosis who scored ≥ 32 on the Autism Quotient. Compared to diagnosed autistic women, probably autistic women had higher empathy and general social functioning, and were more likely to have received a diagnosis of Borderline Personality Disorder. Autistic women had typically received more mental health diagnoses prior to their ASC diagnosis than autistic men. These findings shed light on the history of misdiagnosis experienced by many autistic women.

Cases of undiagnosed autism are thought to be as prevalent in the population as diagnosed autism (i.e., around 1%) (Baron-Cohen et al., 2009), and there is evidence that women are particularly vulnerable to late or missed diagnosis (Baldwin & Costley, 2016; Bancroft, 2012; Begeer et al., 2013; Lai & Baron-Cohen, 2015; Shattuck et al., 2009). According to *Female Phenotype Theory* (FPT), autism in women may be missed by clinicians due to a combination of better-than-expected social functioning, reduced severity of externalising behaviours, and greater internalisation of emotional difficulties (Kopp & Gillberg, 1992; Lai et al., 2011; Mandy et al., 2012). However, because diagnosed women will have exhibited sufficient ‘classic’ autistic traits for their condition to be recognised by clinicians, this cohort may not fully represent the female phenotype, meaning it is crucial to study women who are probably autistic but have never received a formal diagnosis (Halladay et al., 2015). In the present investigation, we aimed to evaluate the female phenotype as contributing to undiagnosed autism in women in two large-scale online surveys.

We have categorised participants according to their self-reported gender identity, rather than their birth ‘sex’, as the current study is concerned with behavioural traits and psychosocial factors rather than physiological factors, and gender identity plays a large role in these (Heidari, et al., 2016). Whilst we have attempted to clearly differentiate between ‘sex’ and ‘gender’ when describing findings from previous studies, most studies did not clarify how participants were defined and so we have referred to ‘sex’ for these.

To date, evidence of a sex difference in the behavioural manifestation of autistic traits has been drawn from diagnosed autistic samples (Lai et al., 2012). In studies of children and adolescents, it has been reported that autistic girls show better reciprocal social conversations, non-verbal communications, and initiating of social interactions than autistic boys (Hiller et al., 2014; Hsiao et al., 2013; McLennan et al., 1993; Rynkiewicz et al., 2016), while demonstrating fewer repetitive and restricted behaviours and interests (RRBIs) (Hiller et al., 2014; Mandy et al., 2012; Ratto et al., 2018). They also display similar levels of friendship quality and motivation to non-autistic girls, while autistic boys show poorer friendship quality and motivation than non-autistic boys (Dean, et al., 2017; Sedgewick et al., 2016). Moreover, autistic women who are not diagnosed until adulthood tend to show less severe socio-communications difficulties and RRBIs on the ADOS (Lai et al., 2011; Wilson et al., 2016), suggesting that their autism went undetected during childhood due to relatively unimpaired social skills. While it has been reported that autistic girls demonstrate similar levels of autistic traits to autistic boys on formal diagnostic assessment measures (e.g., Mussey et al., 2017; Rivet & Matson, 2011), this could indicate that autistic girls are more likely to be socialised to conceal their autistic traits in everyday social situations, a behaviour known as *camouflaging*. Hull, Lai, et al. (2020), Hull, Petrides, et al. (2020)) found that autistic participants who identified their gender as female scored higher on the Camouflaging Autistic Traits Questionnaire (CAT-Q) than those identifying as male. High scores on the CAT-Q indicate greater reliance on behaviours such as ‘masking’ (attempting to conceal autistic traits), ‘compensation’ (attempting to appear ‘non-autistic’), and ‘assimilation’ (wanting to ‘fit-in’).

If many autistic women are indeed not diagnosed due to a distinctly female presentation of autism that is poorly recognised by clinicians, there are likely to be adverse consequences for their mental health. Regardless of sex, autistic adults who received their ASC diagnosis relatively late in life have reported challenges for gaining appropriate support and considerable stress from living so long with an unknown condition (Jones et al., 2001; Stagg & Belcher, 2019). Additionally, the longer autism is missed, the greater the chances that clinicians focus on co-occurring conditions which are common in autism, including anxiety, depression, Obsessive Compulsive Disorder (OCD), and Attention Deficit/ Hyperactivity Disorder (ADHD) (e.g., Russell et al., 2016). There is also a danger of misdiagnosis with conditions that have overlapping features with autism (Lai & Baron-Cohen, 2015). A number of such conditions have been discussed in the literature, including schizophrenia spectrum disorders (Leitman et al., 2014; Aggarwal & Angus, 2015), personality disorders (Fitzgerald, 2005; Hofvander et al., 2009; Lehnhardt et al., 2013; Rabbitte et al., 2017; Ryden et al., 2008), ADHD (Fitzgerald & Corvin, 2001; Gillberg & Billstedt, 2000), OCD (Ivarsson & Melin, 2008; van Steensel et al., 2011), and affective disorders (Fitzgerald & Corvin, 2001; Lehnhardt et al., 2013; Russell et al., 2016). A recent investigation of the clinical histories of 61 autistic adults (22 women, 39 men) found an average 10-year delay between women having first contact with mental health services and being diagnosed with ASC, which was considerably longer than for men (Gesi et al., 2021). This study further found that women were more likely than men to be misdiagnosed at their first evaluation, and that the most common misdiagnosis for women was personality disorder (36.4%). If misdiagnosis results in inappropriate treatment and support, in addition to further delay in gaining an autism diagnosis, then it is likely to exacerbate mental health problems (Kreiser & White, 2014). Moreover, autistic women may suffer emotionally from the strain of trying to conceal their social difficulties through camouflaging (Bargiela et al., 2016). Camouflaging is expected to require valuable cognitive resources, resulting in emotional exhaustion (Livingston et al., 2018). Indeed, high levels of self-reported camouflaging are associated with suicidal thoughts and behaviours, depression, and anxiety (Cassidy et al., 2018; Hull et al., 2019).

In summary, FPT suggests that delayed or missed autism diagnosis in women is driven by a different presentation of autistic traits to that typically shown by men, including less obvious impairments of social functioning and greater internalisation of emotional difficulties (Mandy et al., 2012). Because lack of correct diagnosis is likely to have adverse effects on mental health, it is crucial to learn more about the characteristics of undiagnosed but probably autistic women as a means of improving their chances of timely diagnosis.

In the present investigation we took the novel approach of recruiting a large group of women who appeared to have undiagnosed autism with the goal of building a psychological profile that might facilitate their earlier identification in clinical settings. First, we wanted to evaluate FPT by comparing these probably autistic women with diagnosed autistic women on measures of social functioning. Second, we wanted to document the mental health and history of psychiatric diagnoses of the two groups as a means of gaining insight into reasons for missed ASC diagnosis. Although our main interest was in contrasting the findings for diagnosed- and probably autistic women, we also recruited male participants to enable gender-based comparisons. To screen for undiagnosed autism, we administered the AQ and selected individuals who scored at least 32 but did not have a formal diagnosis of autism. Whilst the AQ is not diagnostic, it is a reliable indicator of autism diagnosis (Baron-Cohen et al., 2001), being 84% accurate in predicting a positive outcome and 78% accurate in predicting a negative outcome in 100 adults referred to an autism assessment centre in the UK (Woodbury-Smith et al., 2005). NICE guidelines also recommend the AQ as part of the Adult Asperger’s Assessment (AAA) (Baron-Cohen et al., 2005), in addition to the Empathy Quotient (EQ: Baron-Cohen & Wheelwright, 2004).

Our investigation comprised two studies with overlapping methods and samples. In Study 1, we administered an online survey of young adults aged 16–40 across the UK that screened for autistic traits using the AQ and collected information about both empathy traits (using the EQ) and formal psychiatric diagnoses (including autism). This age range was targeted to ensure findings were not reflective of historical changes in diagnosis and the introduction of autism subtypes. In Study 2, a follow-up survey assessed the same measures in addition to gauging aspects of social functioning and self-monitoring, anxiety and depression levels, and the ages at which any and each psychiatric diagnosis was received. Our specific research questions and hypotheses were as follows:

1. Do probably autistic women and men have similar scores to diagnosed autistic women and men on the EQ? Based on FPT, it was predicted that probably autistic individuals, particularly women, would score higher on the EQ.
2. Do probably autistic women and men have an advantage in social functioning relative to diagnosed autistic women and men? Based on FPT, it was predicted that probably autistic individuals, particularly women, would demonstrate a stronger motivation for self-monitoring in social situations, which is thought to be important for camouflaging (Estow et al., 2007; Shaffer et al., 1982), as well as superior friendship quality, general social functioning, and theory of mind (ToM).
3. Are probably autistic women and men more prone than diagnosed autistic women and men to receive other psychiatric diagnoses? It was predicted that probably autistic individuals, particularly women, would be more likely to have received psychiatric diagnoses other than autism due to more frequent contact with mental health professionals over the years as they try to cope with undiagnosed ASC, resulting in diagnosis of co-occurring conditions and/or misdiagnosis.
4. Do probably autistic women and men report more anxiety and depression than diagnosed autistic women and men? It was predicted that probably autistic individuals, particularly women, would report higher levels of anxiety and depression due to a combination of the stress of attempting to camouflage autistic traits, the challenges associated with living with an unidentified condition, and possibly coping with a wrong diagnosis.
5. What psychiatric diagnoses did probably autistic women and men receive compared to those with a diagnosis of autism? Given recent evidence, we anticipated that diagnoses of anxiety and mood disorders, as well as personality disorder, would be common for the probably autistic women (Gesi et al., 2021).
6. When do diagnosed autistic women receive other psychiatric diagnoses relative to their autism diagnosis, and how does this compare to diagnosed autistic men? It was predicted that women would tend to receive other psychiatric diagnoses *before* an ASC diagnosis whereas men would tend to receive an ASC diagnosis at an early point in their diagnostic history (see also Gesi et al., 2021).

## Study 1

### Methods

#### Participants

All UK universities were approached for recruitment purposes, given their large and diverse population of young adults. Advertisements were also placed in various media outlets, including local newspapers and social media. Autistic participants were recruited via university disability services, social media, and through autism organisations. Autism was not mentioned in any advertisements targeting non-autistic/undiagnosed participants to avoid attracting mainly those who thought they might be autistic or who had an awareness of the condition. Instead, young adults were invited to help contribute to a nationwide screening study about gender differences in social behaviours and mental health.

There were 5,165 individuals with an average age of 24.92 (*SD* = 6.62) who met the criteria and completed the study (59.1% completion rate). There were 1,324 (25.6%) men and 3,841 (74.4%) women in total, including 26 autistic men and 153 autistic women. Twenty-two participants stated that their current gender was not the same as their birth sex and so they were grouped according to their current gender identity. An additional 126 respondents (95 women and 31 men) who reported a diagnosis of autism were not included in the final sample because we were unable to confirm the diagnosis and their AQ score was lower than the screening cut-off of ≥ 32. Likewise, 23 respondents who identified their gender as ‘other’ were not included in the final sample because their group size was too small for meaningful analysis. Seventy percent of the participants were students, 25% were employed, and 5% were unemployed. Autistic women were diagnosed later, on average, than autistic men (*M* = 23.57, *SD* = 9.34 vs. *M* = 16.92, *SD* = 10.14, respectively). This difference was significant with a medium-to-large effect size, Mann–Whitney U = 1195.00, *p* = 0.003, *d* = 0.68. No other demographic details were collected about participants in this study.

#### Measures

*Autism Quotient (AQ).* The full 50-item Autism Quotient (AQ) (Baron-Cohen et al., 2001) was used to screen participants for a possible ASC. The AQ is reported to have good internal consistency and good test–retest reliability (*r* = 0.7) and a cut off score of ≥ 32 has been found to be accurate in identifying possible cases of ASC in the general population (Baron-Cohen et al., 2001).

*Empathy Quotient (EQ).* The 40-item version of the Empathy Quotient (EQ) (Baron-Cohen & Wheelwright, 2004) was used to assess empathy, which is a key component of successful social interaction (Baron-Cohen et al., 2005). The EQ has excellent test–retest reliability (*r* = 0.97) in both clinical and non-clinical populations (Lawrence et al., 2004). A cut off score of < 30 has been found useful in identifying those with empathy difficulties (Baron-Cohen & Wheelwright, 2004).

*Psychiatric Diagnoses.* Participants were given a checklist containing the common mental health conditions according to the DSM 5 (APA, 2013), including Alcohol/Substance Abuse, Anxiety disorders, Bipolar Disorder, Depression, Eating Disorder, OCD, Personality Disorders, and Schizophrenia. They were asked to select any that they had been diagnosed with by a clinician and were given the opportunity to select ‘other’ if they had a condition not listed. Participants were also asked to indicate whether they had been clinically diagnosed with ASC and, if so, at what age.

#### Procedure

Following the provision of informed consent, participants reported their age, home country, county, employment status, and which psychiatric diagnoses they had, before completing the AQ followed by the EQ. On completion, participants could enter a raffle to win an iPad and were debriefed on the true aims of the research. Participants completed the survey online using Qualtrics, allowing one response per participant, and could not skip items. Ethics approval was obtained from the university research ethics committee (FST/FREP/13/402).

#### Analysis

Participants were grouped by gender and autism status into six categories: women and men reporting a diagnosis of ASC (*diagnosed autistic*), women and men without an ASC diagnosis but scoring above the AQ criteria (≥ 32) (*probably autistic*), and women and men without an ASC diagnosis scoring below the AQ criteria (< 32) (*non-autistic*). Table 1 shows demographic details and sample sizes for each group.

**Table 1:** Participant demographics and descriptive statistics for continuous variables (Study 1)

	Total Sample	Women			Men		
		Diagnosed ASC	Probable ASC	No ASC	Diagnosed ASC	Probable ASC	No ASC
N	5,165	153	690	2,998	26	144	1154
Age (SD)	24.92 (6.62)	27.37 (7.19)	29.17 (6.76)	24.46 (6.45)	25.19 (6.03)	27.58 (7.21)	22.93 (5.43)
Other Psychiatric Diagnoses		1.56 (1.58)	1.12 (1.40)	0.47 (0.89)	1.12 (1.28)	0.62 (1.03)	0.31 (0.74)
Psychiatric diagnosis (%)		Affective disorder: 63.4	Affective disorder: 51.9	Affective disorder: 25.2	Affective disorder: 46.2	Affective disorder: 26.4	Affective disorder: 12.9
		OCD: 8.5	OCD: 6.1	OCD: 1.8	OCD: 15.4	OCD: 2.1	OCD: 1.6
		Eating disorder: 11.8	Eating disorder: 6.8	Eating disorder: 4.1	Eating disorder: 3.8	Eating disorder: 1.4	Eating disorder: 0.6
		Substance abuse: 3.3	Substance abuse: 2	Substance abuse: 0.8	Substance abuse: 3.8	Substance abuse: 2.1	Substance abuse: 1.1
		BPD: 3.3	BPD: 4.1	BPD: 0.7	BPD: 3.8	BPD: 0	BPD: 0.4
		Schizoid PD: 0	Schizoid PD: 0.1	Schizoid PD: 0.1	Schizoid PD: 3.8	Schizoid PD: 0.7	Schizoid PD: 0.3
		Schizophrenia: 0.7	Schizophrenia: 0.4	Schizophrenia: 0.1	Schizophrenia: 0	Schizophrenia: 0.7	Schizophrenia: 0.2
		Other: 7.8	Other: 4.9	Other: 2.6	Other: 3.8	Other: 6.9	Other: 1.8
Employment (%)		Student: 49.6	Student: 36.1	Student: 74.2	Student: 61.6	Student: 51.4	Student: 85.7
		Employed: 28.8	Employed: 49.1	Employed: 22.3	Employed: 19.2	Employed: 44.5	Employed: 12.9
		Unemployed/retired: 21.6	Unemployed/retired: 14.8	Unemployed/retired: 3.4	Unemployed/retired: 19.2	Unemployed/retired: 4.2	Unemployed/retired: 1.4
Age of ASC		23.57 (9.34)	N/A	N/A	16.92 (10.14)	N/A	N/A
AQ total (SD)	22.47 (9.42)	39.75 (4.38)	36.52 (3.79)	18.89 (6.89)	39.50 (4.22)	36.05 (3.39)	19.00 (6.11)
EQ total (SD)	38.58 (14.93)	19.13 (8.56)	23.19 (10.25)	44.37 (12.93)	16.54 (6.94)	19.55 (9.05)	38.17 (12.02)

*SD* standard deviation, *ASC* autism spectrum condition, *AQ* Autism Quotient, *EQ* Empathy Quotient, *OCD* Obsessive Compulsive Disorder, *ADHD* Attention Deficit Hyperactivity Disorder, *BPD* Borderline Personality Disorder, *PD* Personality Disorder

Due to the expected departures from normality on the AQ and EQ given group allocation and unequal group sizes, non-parametric tests were employed. The Kruskal–Wallis H was used to examine group differences in age and EQ scores, with Bonferroni-corrected Mann–Whitney U tests for pairwise comparisons, adjusting the *p* value criteria to < 0.017. Chi-Square analyses assessed differences in the frequency of other psychiatric diagnoses across groups using the Bonferroni-corrected *p* value of < 0.017. Probably autistic women were slightly older on average than diagnosed autistic women (*M* = 29.17, *SD* = 6.759 vs. *M* = 27.37, *SD* = 7.193 respectively), U = 45,024.50, *p* < 0.001, *d* = 0.26. Likewise, probably autistic men were slightly older than diagnosed autistic men (*M* = 27.58, *SD* = 7.210 vs. *M* = 25.19, *SD* = 6.027 respectively), but the difference was not significant, *p* > 0.05. These findings suggest that participants in the probably autistic groups did not lack an ASC diagnosis simply because they had less time to acquire one.

### Results and Discussion

*EQ.* Due to the expected negative association between the AQ and EQ, and because participants were grouped according to their AQ scores, we compared only the diagnosed and probably autistic groups on the EQ. Probably autistic women scored significantly higher than diagnosed autistic women, U = 40,045.50, *p* < 0.001, *d* = 0.43, but EQ scores did not differ significantly between probably autistic men and diagnosed autistic men, U = 1527.50, *p* = 0.136, *d* = 0.37. Gender comparison within each diagnostic group revealed no significant difference in EQ scores between diagnosed autistic men and women, U = 1651.00, *p* = 0.166. However, a significant difference with a small effect size was found between probably autistic men and women, U = 3964.30, *p* < 0.001, *d* = 0.38, with probably autistic women scoring higher. Similarly, there was a significant difference with a medium effect size between non-autistic men and women, U = 1,244,328.00, *p* < 0.001, *d* = 0.50, with non-autistic women scoring higher.

*Psychiatric Diagnoses other than Autism.* There were significant differences between diagnostic groups in the number of other psychiatric diagnoses made for women: *X* ^2^(2) = 282.074, *p* < 0.001. Diagnosed autistic women had significantly more psychiatric diagnoses on average than probably autistic women, U = 44,715.00, *p* = 0.002, *d* = 0.29, and both groups had significantly more diagnoses than non-autistic women, *p*’s < 0.001, *d* = 0.85 and *d* = 0.55 respectively. Significant differences were also observed between diagnostic groups for men, *X* ^2^(2) = 40.163, *p* < 0.001. However, diagnosed and probably autistic men had a similar number of psychiatric diagnoses after Bonferroni correction, U = 1417.50, *p* = 0.025, *d* = 0.43, while both groups had significantly more than non-autistic men, *p*’s < 0.001, *d* = 0.77 and *d* = 0.35 respectively. Gender comparison within each diagnostic group revealed no significant difference in the number of other psychiatric diagnoses between diagnosed autistic men and women, U = 1630.00, *p* = 0.128. However, a significant difference with a medium effect size was found between probably autistic men and women, U = 36,846.00, *p* < 0.001, *d* = 0.41, with probably autistic women scoring higher. Similarly, there was a significant difference with a small effect size between non-autistic men and women, U = 1,520,050.50, *p* < 0.001, *d* = 0.20, with non-autistic women scoring higher.

We next explored group differences in the incidence of specific diagnoses. No such analyses are reported for alcohol/substance abuse, eating disorders, Schizophrenia or Schizoid PD, or for men, as the frequency counts were too low.

*BPD.* As can be seen in Table 1, probably autistic women were more likely to have a diagnosis of BPD than either diagnosed autistic women (OR: 1.25; 95% CI 0.48, 3.30) or non-autistic women (OR: 0.18; 95% CI 0.10, 0.31). Diagnosed autistic women were more likely to have a BPD diagnosis than non-autistic women (OR: 0.22, 95% CI 0.08, 0.59). The difference between groups was significant, *X*^2^(2) = 47.719, *p* < 0.001, φ = 0.111.

*OCD.* Diagnosed autistic women were more likely to have an OCD diagnosis than either probably autistic women (OR: 0.70; 95% CI 0.37, 1.34) or non-autistic women (OR: 0.19; 95% CI 0.10, 0.36), and probably autistic women were more likely to have an OCD diagnosis than non-autistic women (OR: 0.28; 95% CI 0.18, 0.42). The difference between groups was significant, *X*^2^(2) = 57.135, *p* < 0.001, φ = 0.122.

*Affective Disorder.* Diagnosed autistic women were more likely to have an affective disorder diagnosis than either probably autistic women (OR: 0.62; 95% CI 0.43, 0.89) or non-autistic women (OR: 0.20; 95% CI 0.14, 0.27), and probably autistic women were more likely to have an affective disorder diagnosis than non-autistic women (OR: 0.31; 95% CI 0.26, 0.37). The difference between groups was significant, *X*^2^(2) = 259.745, *p* < 0.001, φ = 0.260.

*Eating Disorders.* Diagnosed women were more likely to have been diagnosed with an eating disorder than either probably autistic women (OR: 0.55; 95% CI 0.31, 0.97) or non-autistic women (OR: 0.32; 95% CI 0.19, 0.55), and probably autistic women were more likely to have been diagnosed with an eating disorder than non-autistic women (OR: 0.59; 95% CI 0.42, 0.84). The difference between groups was significant, *X*^2^(2) = 24.54, *p* < 0.001, φ = 0.0.8.

In summary, and as expected, Study 1 found that women who are probably autistic scored higher on the EQ than diagnosed autistic women. Contrary to predictions, though, we did not find that probably autistic women were more likely than diagnosed autistic women to have other psychiatric diagnoses; instead, the rate of psychiatric diagnoses was higher in the diagnosed group. This pattern of findings was not replicated among the male participants, as probably autistic men scored similarly to diagnosed autistic men on the EQ and had a similar prevalence of other psychiatric diagnoses. Finally, while diagnosed autistic women were more likely to report diagnoses of OCD, affective disorders, and eating disorders, probably autistic women were more likely to have a BPD diagnosis. In Study 2, we followed up these findings using a wider range of measures of social functioning. Additionally, we measured levels of depression and anxiety, and asked participants their age when receiving each of their psychiatric diagnoses.

## Study 2

### Methods

#### Participants

All Study 1 participants who consented to be re-contacted were approached in the first instance. Further, advertisements were circulated for new participants who fulfilled the eligibility criteria. Again, advertisements did not mention autism but instead called for participants to take part in a study looking at ‘gender differences in social awareness and motivation’.

A total of 505 people who met the inclusion criteria completed the survey (51.04% completion rate), of whom 372 had participated in Study 1. Eight participants who identified with ‘other’ or unknown gender identities were not included in the final sample due to their small group size. Additionally, 23 diagnosed autistic participants were not included in the final sample due to scoring below the criterion value of 32 on the AQ (11 men and 12 women). The final sample comprised 103 men and 402 women; of these, there were 90 diagnosed autistic women and 27 diagnosed autistic men. The average age of participants was 27.30 (*SD* = 6.00). On average, autistic women were diagnosed with ASC later than autistic men (*M* = 24.88, *SD* = 7.89 vs. *M* = 18.96, *SD* = 10.95), U = 793.500, *p* = 0.008, *d* = 0.62. Most participants were white British (94.5%), with 44.4% being students, 44.1% in employment, and 11.7% unemployed. Demographic information and descriptive statistics for each of the six groups are presented in Table 2.

**Table 2:** Participant demographics and descriptive statistics for continuous variables (Study 2)

	Total Sample	Women			Men		
		Diagnosed ASC	Probable ASC	No ASC	Diagnosed ASC	Probable ASC	No ASC
N	505	90	77	235	27	9	67
Age (SD)	27.30 (6.0)	28.84 (6.19)	30.56 (5.82)	26.24 (5.57)	26.56 (6.22)	26.67 (7.52)	25.42 (5.25)
Age of ASC		24.88 (7.89)	N/A	N/A	18.95 (2.11)	N/A	N/A
AQ total (SD)	27.11 (11.24)	39.67 (5.13)	37.13 (4.10)	19.30 (7.35)	37.93 (4.86)	35.22 (2.22)	19.76 (6.43)
EQ total (SD)	33.12 (15.65)	18.79 (8.23)	22.27 (10.17)	43.91 (12.34)	17.33 (7.98)	19.67 (6.04)	36.19 (11.10)
FQ total (SD)	68.37 (23.86)	54.88 (21.35)	53.06 (18.36)	81.21 (19.84)	54.48 (25.80)	40.33 (13.64)	67.15 (19.94)
SMS total (SD)	11.67 (4.42)	10.18 (4.94)	10.45 (4.42)	12.25 (3.92)	9.67 (4.38)	11.89 (3.06)	14.28 (3.85)
RMET total (SD)	7.85 (1.81)	6.94 (2.40)	7.64 (1.91)	8.31 (1.41)	6.78 (2.03)	7.56 (2.35)	8.27 (0.99)
SFS total (SD)	131.74 (24.60)	116.64 (26.24)	125.53 (20.29)	141.01 (21.48)	113.63 (19.22)	121.33 (13.99)	136.22 (24.51)
SFS Engagement	101.83 (11.24)	94.82 (7.29)	99.34 (9.81)	106.33 (11.29)	94.48 (6.33)	93.33 (8.01)	102.84 (11.59)
SFS Interpersonal	129.82 (16.16)	121.10 (15.08)	123.5 (16.09)	135.26 (13.91)	118.26 (15.60)	126.56 (19.45)	134.34 (15.50)
SFS Independence Competence	113.57 (11.86)	103.34 (13.41)	112.60 (12.33)	117.76 (8.50)	102.35 (12.23)	114.06 (5.18)	118.71 (7.79(
SFS Independence Performance	111.06 (13.77)	104.97 (15.91)	109.23 (13.10)	115.81 (11.55)	101.85 (10.99)	103.83 (7.59)	109.87 (13.88)
SFS Prosocial	111.60 (13.31)	105.24 (13.52)	105.53 (10.43)	115.76 (12.17)	106.37 (10.75)	107.33 (9.27)	115.55 (15.03)
SFS Recreation	111.39 (15.71)	110.92 (16.90)	112.19 (15.17)	112.43 (15.99)	106.80 (13.34)	109.17 (12.97)	109.72 (14.98)
SFS Employment	104.20 (16.34)	101.18 (17.18)	103.71 (16.65)	106.55 (15.43)	99.90 (17.82)	98.75 (19.92)	108.72 (14.16)
PHQ-9	10.31 (6.57)	14.40 (6.30)	12.34 (6.46)	8.31 (1.41)	12.41 (5.76)	10.22 (4.68)	7.24 (5.95)
GAD-7	8.43 (6.11)	11.93 (6.02)	10.57 (5.94)	7.20 (5.65)	10.22 (4.87)	6.00 (3.71)	4.93 (5.24)

*SD* standard deviation, *ASC* autism spectrum condition, *AQ* Autism Quotient, *EQ* Empathy Quotient, *FQ* Friendship Quotient, *SMS* Self-Monitoring Scale, *RMET* Reading the Mind in the Eyes Test, *SFS* Social Functioning Scale, *PHQ* Patient Health Questionnaire, *GAD* Generalised Anxiety Disorder

#### Measures

The AQ and EQ were presented to new participants only, with the following additional measures presented to all participants:

*Self-Monitoring Scale (SMS):* The SMS is a 25-item scale requiring a ‘yes’ or ‘no’ response on each item (Snyder, 1974). This scale assesses the self-control of expressive behaviours, which requires the ability to monitor one’s own inner state and the social situations one is in, and to change and monitor one’s own behaviour accordingly. Previously established norms show that scores of 15–22 are high, 9–14 are intermediate, and 0–8 are low (Ickes & Barnes, 1977). The scale has good reliability (*r* = 0.70) and test–retest reliability (0.83) (Snyder, 1974).

*The Friendship Questionnaire (FQ):* The FQ is a 35-item scale (27 of which are scored) measuring the quality of participants’ friendships and relationships, which constitutes an important part of social functioning (Baron-Cohen & Wheelwright, 2003). The questionnaire employs a range of responses from Likert scales to rankings, with a maximum score of 135. Higher scores indicate that the respondent values close, empathic, supportive, and caring friendships, and that they enjoy the company of people and interacting with others for its own sake. Baron-Cohen and Wheelwright (2003) found the internal consistency of the scale was excellent, with Chronbach’s alpha ranging from 0.75 to 0.84.

*Social Functioning Scale (SFS):* Birchwood et al.’s (1990) SFS is a 79-item, 7-factor self-report assessment based on the impairments and disability assessed by the Disability Assessment Schedule (Ustan et al., 2010). It measures social engagement/withdrawal, interpersonal behaviour, pro-social activities, recreation, independence-competence, independence-performance, and employment/occupation. The SFS has good reliability (*r* = 0.80) and good internal consistency as demonstrated by item-total correlations (*r* = 0.71). Factor analyses revealed that it is appropriate to calculate a mean score across the whole SFS, as well as for individual factors (Birchwood, 1990).

*Reading the Mind in the Eyes Test – brief version (RMET):* The brief version of the RMET (Olderbak et al., 2015), initially developed by Baron-Cohen et al. (2001), was used to measure ToM. The original RMET was designed to differentiate between various clinical populations (mainly autistic people) and non-autistic controls in terms of ToM capabilities. The brief version presents participants with 10 images of other peoples’ eyes and provides a choice of four labels to choose from which could describe the person’s mental state. Internal consistency for this test is good (α = 0.73).

*The Patient Health Questionnaire – 9 (PHQ-9):* The PHQ-9 measures depression using the nine DSM-IV criteria (Kroenke et al., 2001). Participants rate each item according to how often they experience the symptom from ‘not at all’ to ‘every day’. The internal reliability of the scale is excellent, with Chronbach’s alpha ranging from 0.84 to 0.89. The PHQ-9 also has excellent test–retest reliability (*r* = 0.84) and good construct validity, with scores on the scale strongly associated with functional status, disability days, and symptom-related difficulty. The scale has been validated in autistic adults and demonstrates high reliability in this cohort (α = 0.91) (Arnold et al., 2020).

*Generalized Anxiety Disorder – 7 (GAD-7):* The GAD-7 scale has seven items derived from the DSM-IV symptom criteria for GAD and from other existing anxiety scales (Spitzer et al., 2006). Participants rate each item as to how often they experience the symptom from ‘not at all’ to ‘every day’. The internal reliability of the scale is excellent, with a Chronbach’s alpha of 0.92, and it has good test–retest reliability (*r* = 0.83). The scale also has strong construct validity, with scores associating strongly with scores from a functioning scale, and convergent validity, with scores on the scale correlating strongly with two other anxiety scales (Spitzer et al., 2006). Studies using the scale in autistic populations have reported high reliability (α = 0.92) (Hull et al., 2019).

#### Procedure

The survey design and procedure were the same as for Study 1. Returning participants entered an individualised password emailed to them, allowing them to skip the AQ and EQ; all other participants completed these first. These were followed by the FQ, SMS, SFS, RMET, PHQ-9, and GAD-7, in that order. Demographic data were then collected including age, gender, country of birth, county, ethnicity, employment status, psychiatric diagnoses, and age at which each psychiatric diagnosis was received. Participants could enter a raffle for a £100 Amazon voucher on completion of the survey.

#### Analysis

Groupings were the same as Study 1. Group differences on the EQ, mental health measures (PHQ-9 and GAD-7), and the social functioning measures (FQ, SMS, SFS, and RMET) were investigated with Kruskal–Wallis H, followed by Bonferroni-corrected Mann–Whitney U tests for pairwise comparisons, adjusting the *p* value criteria to < 0.017. No comparisons were carried out with probably autistic men due to the small sample size (*n* = 9). Finally, to situate the age of ASC diagnosis within the context of all other psychiatric diagnoses, for all diagnosed autistic participants the following two variables were calculated and compared using Wilcoxon tests: (1) the number of psychiatric diagnoses prior to ASC diagnosis, and (2) the number of psychiatric diagnoses following the ASC diagnosis. In the rare cases where another psychiatric diagnosis was concurrent with the ASC diagnosis, only the latter was counted. Additionally, ASC diagnosis was coded as only-, first-, middle- or last diagnosis, and compared for diagnosed ASC men and women using chi-square.

Preliminary analyses confirmed that there was no significant difference in age between the diagnosed autistic women and the probably autistic women in this sample (*M* = 27.37, *SD* = 7.19 vs. *M* = 29.17, *SD* = 6.76, respectively), or between the diagnosed autistic men and probably autistic men (*M* = 25.19, *SD* = 6.03 vs. *M* = 27.58, *SD* = 7.21): all *p*’s > 0.06.

### Results and Discussion

*EQ:* Like Study 1, diagnosed autistic women scored significantly lower than probably autistic women on the EQ, U = 40,045.50, *p* < 0.001, *d* = 0.38. This difference was not observed between diagnosed autistic men and probably autistic men, U = 92.50, *p* = 0.295, *d* = 0.33. Gender comparison within each diagnostic group revealed no significant difference in EQ scores between diagnosed autistic men and women, U = 1086.00, *p* = 0.403. However, there was a significant difference with a medium effect size between non-autistic men and women, U = 5144.500, *p* < 0.001, *d* = 0.67, with non-autistic women scoring higher.

#### Social Functioning

*SMS:* There was a significant difference in self-monitoring between female diagnostic groups, *X* ^2^(2) = 18.832, *p* < 0.001. Diagnosed autistic and probably autistic women did not differ, with both groups having significantly lower scores than non-autistic women, *p* = 0.001, *d* = 0.46, and *p* = 0.005, *d* = 0.43, respectively. Group mean scores did not differ on the other-directedness subscale, *X* ^2^(2) = 0.404, *p* = 0.817, but there were significant differences on both the acting subscale, *X*^2^(2) = 15.50, *p* < 0.001, and extraversion subscale, *X*^2^(2) = 71.577, *p* < 0.001. Diagnosed autistic and probably autistic women scored similarly on the acting subscale and both groups had significantly lower scores than non-autistic women, *p* = 0.017, *d* = 0.35 and *p* = 0.009, *d* = 0.39 respectively. Diagnosed autistic women and probably autistic women also scored similarly on the extraversion subscale, and both groups had significantly lower scores than non-autistic women, *p* < 0.001, *d* = 0.93, and *p* < 0.001, *d* = 0.82, respectively.

Group differences on the total SMS were also observed for men, *X* ^2^(2) = 19.740, *p* < 0.001, and diagnosed autistic men had significantly lower scores than non-autistic men, *p* = 0.001, *d* = 1.12. Gender comparison within each diagnostic group revealed no significant difference on the SMS between diagnosed autistic men and women, U = 1135.50, *p* = 0.606. However, there was a significant difference with a medium effect size between non-autistic men and women, U = 5621.50, *p* < 0.001, *d* = 0.52, with non-autistic women scoring higher.

*FQ:* There was a significant difference in friendship scores between female diagnostic groups, *X* ^2^(2) = 115.419*, p* < 0.001. Diagnosed autistic and probably autistic women scored similarly, and both groups had significantly lower scores than non-autistic women, *p*’s < 0.001, *d* = 1.23 and *d* = 1.47, respectively. Differences were also observed between male diagnostic groups, *X* ^2^(2) = 13.732*, p* = 0.001, however, diagnosed autistic men scored similarly to non-autistic men. Gender comparison within each diagnostic group revealed no significant difference in FQ scores between diagnosed autistic men and women, U = 903.00, *p* = 0.893. However, there was a significant difference with a large effect size between non-autistic men and women, U = 4510.50, *p* < 0.001, *d* = 0.71, with non-autistic women scoring higher.

*SFS:* There was a significant difference in social functioning between female diagnostic groups on the total SFS, *X* ^2^(2) = 74.404, *p* < 0.001. Diagnosed autistic women had a significantly lower mean SFS score than probably autistic women, although this was not significant when Bonferroni corrections were applied, *p* = 0.025, *d* = 0.38. However, the effect size was medium and both groups had significantly lower scores than non-autistic women, *p*’s < 0.001, *d* = 1.02 and *d* = 0.74, respectively. Differences were also observed between male diagnostic groups on the total SFS, *X* ^2^ (2) = 19.702*, p* < 0.001, with diagnosed autistic men scoring significantly lower than non-autistic men, *p* = 0.02, *d* = 0.75. Gender comparison within each diagnostic group revealed no significant difference in SFS scores between diagnosed autistic men and women, U = 1095.00, *p* = 0.437, or between non-autistic men and women, U = 7075.50, *p* = 0.206.

For women, significant group differences were also found for the majority of SFS subscales when examined individually (the same analyses were not conducted for men due to small group sizes). There was a significant difference on the engagement subscale between groups, *X* ^2^(2) = 78.702, *p* < 0.001. Diagnosed autistic women scored significantly lower on average than probably autistic women, and both groups scored lower than non-autistic women. There was a significant difference on the interpersonal communication subscale between groups, *X*^2^(2) = 65.497, *p* < 0.001. Diagnosed autistic women scored similarly to probably autistic women but lower than non-autistic women. There was a significant difference on the independence-performance subscale between groups, *X* ^2^(2) = 39.821, *p* < 0.001. Diagnosed autistic women scored similarly to probably autistic women but lower than non-autistic women. There was a significant difference on the independence-competence subscale between groups, *X* ^2^(2) = 89.276, *p* < 0.001. Diagnosed autistic women scored significantly lower than probably autistic women, and both groups scored lower than non-autistic women. There was a significant difference on the prosocial subscale between groups, *X* ^2^(2) = 63.834, *p* < 0.001. Diagnosed autistic women scored similarly to probably autistic women but lower than non-autistic women. There was a significant difference on the employment subscale between groups, *X* ^2^(2) = 31.875, *p* < 0.001. Probably autistic and non-autistic women scored similarly, but higher than diagnosed autistic women. Finally, there was no significant difference between groups on the recreation subscale, *X* ^2^(2) = 0.618, *p* = 0.734.

*RMET:* There was a significant difference in ToM between female diagnostic groups, *X* ^2^(2) = 24.543, *p* < 0.001. Diagnosed and probably autistic women scored similarly, while both groups had significantly lower scores than non-autistic women, *p* < 0.001, *d* = 0.71, and *p* = 0.007, *d* = 0.41, respectively. There was also a significant difference in ToM between male diagnostic groups, *X* ^2^(2) = 11.374, *p* = 0.003, with diagnosed autistic men scoring significantly lower than non-autistic men, *p* = 0.001, *d* = 0.93. Gender comparison within each diagnostic group revealed no significant difference in the RMET scores between diagnosed autistic men and women, U = 1115.00, *p* = 0.513, or between non-autistic men and women, U = 7251.00, *p* = 0.309.

#### Mental Health

*GAD7:* There was a significant difference in anxiety between female diagnostic groups, *X*^2^(2) = 47.328, *p* < 0.001. Diagnosed and probably autistic women scored similarly, while both groups had significantly higher scores than non-autistic women, *p*’s < 0.001, *d* = 0.81 and *d* = 0.58 respectively. Significant differences in anxiety scores were also observed between male diagnostic groups, *X* ^2^(2) = 21.327, *p* < 0.001, with diagnosed men scoring significantly higher than non-autistic men, *p* < 0.001, *d* = 0.58. Gender comparison within each diagnostic group revealed no significant difference in the GAD-7 scores between diagnosed autistic men and women, U = 1024.50, *p* = 0.217. However, there was a significant difference with a medium effect size between non-autistic men and women, U = 5735.00, *p* < 0.001, *d* = 0.42, with non-autistic women scoring higher.

*PHQ-9:* There was a significant difference in PHQ-9 scores between female diagnostic groups, *X* ^2^(2) = 55.509, *p* < 0.001. Diagnosed and probably autistic women scored similarly, while both groups had significantly higher scores than non-autistic women, *p*’s < 0.001, *d* = 0.91 and *d* = 0.57, respectively. Significant differences in depression scores were also observed between male diagnostic groups, *X* ^2^(2) = 16.011, *p* < 0.001, with diagnosed autistic men scoring significantly higher than non-autistic men, *p* < 0.001, *d* = 0.81. Gender comparison within each diagnostic group revealed no significant difference in the PHQ-9 scores between diagnosed autistic men and women, U = 998.50, *p* = 0.161, or between non-autistic men and women, U = 6594.00, *p* = 0.42.

*Age of other psychiatric diagnoses:* For diagnosed autistic women, the number of other psychiatric diagnoses received prior to the ASC diagnosis (*M* = 1.74, *SD* = 1.41) was significantly greater that the number of psychiatric diagnoses received afterwards (*M* = 0.40, *SD* = 0.92), *z* = -4.798, *p* < 0.001, *d* = 1.23. For diagnosed autistic men, in contrast, the number of other psychiatric diagnoses received prior to the ASC diagnosis (*M* = 0.80, *SD* = 0.86) was not significantly different to the number of psychiatric diagnoses received afterwards (*M* = 0.53, *SD* = 0.52), z = -0.714, *p* = 0.475, *d* = 0.38.

For women, the ASC diagnosis was the last diagnosis on 51 of 89 occasions (57%). In contrast, for men the ASC diagnosis was the last on 7 of 27 occasions (26%). Chi-Square analysis revealed a significant difference between men and women, *X*^2^(2) = 9.137, *p* = 0.028, φ = 0.281. Diagnosed autistic women were 3.8 times more likely than diagnosed autistic men to have received their autism diagnosis last.

In summary, Study 2 replicated Study 1’s finding that probably autistic women scored higher on the EQ than diagnosed autistic women. As predicted, probably autistic women also scored higher in terms of general social functioning, particularly in terms of engagement, independence/competence, and employment. However, the groups reported equivalent levels of depression and anxiety and attained similar mean scores for self-monitoring, friendship quality, and theory of mind. No statistical comparisons were possible involving the probably autistic men due to the underpowered sample, but diagnosed autistic men scored significantly lower than non-autistic men in terms of social functioning, and significantly higher than non-autistic men in terms of depression and anxiety.

For diagnosed autistic women and men, the results confirmed hypotheses in showing that (1) women tended to receive most of their other psychiatric diagnoses prior to their ASC diagnosis whereas men did not, and (2) for women, the ASC diagnosis was usually the final diagnosis, whereas for men it was not.

## General Discussion

Previous evaluations of FPT compared the behavioural manifestations of autism between diagnosed autistic women and diagnosed autistic men. Because diagnosed autistic women will have presented with sufficient ‘classic’ autistic traits for their autism to be recognised by clinicians, they may not fully represent the female phenotype. In the present investigation, we took the novel approach of recruiting a large sample of men and women with high levels of autistic traits who lacked an ASC diagnosis and compared them with diagnosed autistic men and women on measures of empathy (Studies 1 and 2) and social functioning (Study 2). We also compared the groups for the incidence of psychiatric diagnoses other than autism (Study 1), participants’ history of psychiatric diagnoses, and current mental health (Study 2). We aimed to evaluate FPT as an explanation of delayed ASC diagnosis in women, and to shed light on the mental health and typical psychiatric trajectories of this population.

Based on FPT, our first prediction was that probably autistic women would have higher scores on the EQ than diagnosed autistic women. This hypothesis was confirmed in both studies. Importantly, this same pattern was not observed for men, and probably autistic women had significantly higher EQ scores than probably autistic men, suggesting a distinct female advantage in the empathy domain. Similarly, and as expected, probably autistic women achieved higher scores than diagnosed autistic women for general social functioning as gauged by the SFS. This was particularly apparent for the engagement and independence-competence subscales of the SFS and evidenced through comparable employment scores to non-autistic women. Nevertheless, social impairments were reported by the probably autistic women, and they scored significantly lower than non-autistic women but equivalently to diagnosed autistic women on subscales of the SFS gauging interpersonal behaviour, pro-social activities, recreation, and independence-performance.

However, other aspects of our findings were not as anticipated. First, we found no evidence that probably autistic women had stronger friendship motivation and quality than diagnosed autistic women. Likewise, Baron-Cohen and Wheelwright (2003) failed to find a difference on the FQ between diagnosed autistic women and diagnosed autistic men, suggesting that quality of friendships might not be a good indicator of the female phenotype of autism. Given that probably autistic women do have social impairments, as described above, friendship may remain a difficult aspect of socialising for many to manage. Indeed, regardless of whether they have an ASC diagnosis, autistic women may find themselves ostracised by non-autistic people due to their social differences (Belcher, et al., 2021). Second, probably autistic women showed equivalent scores to diagnosed autistic women for both ToM and self-monitoring. The lack of any group difference in ToM might be considered surprising given the positive relationship between empathising and ToM (e.g., Stietz et al., 2019), but does reinforce the earlier conclusion that, despite their empathy advantage, probably autistic women experience many of the social difficulties exhibited by diagnosed autistic women. No previous study has evaluated self-monitoring in the autistic population, but self-monitoring has been linked with the ability to adjust effectively to different social situations and to socially mimic others (Estow et al., 2007; Shaffer et al., 1982; Snyder, 1974). More recent measures of camouflaging intent such as the Camouflaging Autistic Traits Questionnaire (CAT-Q; Hull et al., 2019), which ask participants directly about their deliberate efforts to mask autistic traits and which were published after the current study took place, have observed greater levels in autistic individuals than non-autistic individuals (Belcher et al., 2021; Hull, Lai, et al., 2020; Hull, Petrides, et al., 2020). In contrast, self-monitoring scores in the present study were significantly *lower* for the autistic participants than the non-autistic participants, indicating that the SMS is picking up aspects of self-presentation in social situations that are quite different from those used to mask perceived autistic traits. Given this conclusion, it would make sense in future research to compare diagnosed autistic individuals with probably autistic individuals on the CAT-Q rather than the SMS.

If a distinctly female phenotype of autism exists, then it likely reflects both biological and social influences. For example, women might have an evolutionary edge in empathising and social behaviours compared to men (e.g., Baron-Cohen, 1995) but also experience stronger socialisation pressures to develop competence in these domains (Bem, 1974). Interestingly, an empathy advantage was also displayed by non-autistic women relative to non-autistic men, and they scored significantly higher for both self-monitoring and friendship quality. While these findings hint at a general female superiority in empathising and aspects of social functioning that might make autism less obvious when it occurs in women, it cannot be ruled out that the gender difference was more to do with reporting of behaviours rather than the behaviours per se. That is, the internalisation of gender norms may lead women to rate themselves higher on scales that pertain to empathising and social behaviours. It has been observed that autistic women tend to give higher ratings than autistic men on AQ items, which could indicate heightened awareness of traits deemed less socially acceptable for women (Lehnhardt et al., 2016; Lai et al., 2013). In future research, therefore, it would be advisable to assess empathy and social functioning via measures other than self-report.

In terms of mental health ratings, our finding that GAD-7 and PHQ-9 results were similar between diagnosed- and probably autistic women is at odds with our hypothesis that the former group would experience more anxiety and depression from attempting to camouflage their social difficulties and/or lack of proper diagnosis and support. In explaining this null outcome, it is important to acknowledge that the diagnosed women in our sample received their autism diagnosis at a relatively late age (i.e., 27–28 years on average), meaning there was probably much overlap between these women and the probably autistic women in how their autism presented. It is also worth noting that while camouflaging has been shown to negatively affect mental health (Cassidy et al., 2018; Hull et al., 2019), other factors, such as autistic acceptance from others and oneself, can predict depression in autistic adults (Cage et al., 2018). Indeed, sensory, emotional, and cognitive factors have all been found to contribute to anxiety in autistic people. For example, South and Rodgers (2017) proposed a model that included sensory functioning, intolerance for uncertainty, and alexithymia (problems reading one’s own emotions) as key contributing factors in the development of anxiety in this population.

In terms of other psychiatric diagnoses, it was predicted that probably autistic women would report a greater number of these than diagnosed autistic women, due to a history of being labelled by mental health professionals with co-occurring or overlapping conditions. This was not found to be the case and, on average, diagnosed autistic women had more psychiatric diagnoses than probably autistic women. Nevertheless, probably autistic women reported significantly more psychiatric diagnoses than probably autistic men, and the prediction that probably autistic women would be more likely to have diagnoses that could be classed as differential diagnoses due to overlapping features with ASC was partially supported. Specifically, while diagnosed autistic women were more likely to have diagnoses of affective disorders, OCD, and eating disorders, probably autistic women were more likely to have a diagnosis of BPD. This supports the suggestion that clinicians often diagnose BPD in preference to ASC due to strong similarity in symptoms (Bargiela et al., 2016; Gesi et al., 2021; Lai & Baron-Cohen, 2015; Rabbitte et al., 2017; Ryden et al., 2008). For example, both autistic women and women with BPD demonstrate difficulties in relationships and regulating their emotions, as well as impulsivity and stress-related paranoid ideation (Fitzgerald, 2005). With classic signs of autism masked, such as RRBIs and socio-communication problems, clinicians may favour diagnosing BPD, which is more commonly seen in women in the general population (APA, 2000). Whilst it was against our expectation that diagnosed autistic women had more of the other types of differential diagnoses, this may reflect the fact that diagnosed autistic women would be well known to mental health services.

Finally, the current study explored the history of psychiatric diagnoses for diagnosed autistic participants by comparing the ages at which different diagnoses were received. As hypothesized, diagnosed autistic women gained significantly more psychiatric diagnoses prior to their ASC diagnosis compared to afterwards. For diagnosed autistic men, in contrast, there was no significant difference in the number of psychiatric diagnoses gained before versus after their autism diagnosis. Accordingly, the ASC diagnosis was much more likely to come last for women than for men. These findings support the suggestion that ASC diagnosis is commonly delayed for autistic women due to clinicians’ focus on other conditions (Gesi et al., 2021; Lai & Baron-Cohen, 2015).

## Limitations

The present study contributes important new knowledge regarding the psychological profile of women who are probably autistic, and their history of psychiatric diagnoses, but is not without limitations. First, our sample included an unusually high percentage of diagnosed and probably autistic people. In Study 1, 3.98% of the women and 1.96% of the men were diagnosed autistic, while 17.96% the women and 10.88% of the men were probably autistic. In contrast, current prevalence estimates are that 1% of the general adult population has an ASC diagnosis and a further 1% is potentially undiagnosed (Baron-Cohen et al., 2001; Russell, 2014). These figures suggest that the measures taken to avoid selection bias in our study were unsuccessful, and that we unduly attracted autistic participants and those who thought they might be autistic but did not yet have a diagnosis, particularly women. This could have occurred because participants spread word about our research after receiving their debriefing, and thus future survey studies of this nature should delay providing any feedback until all the data have been collected. It would also be beneficial to ask probably autistic participants in the study to indicate whether or not they suspect they may be autistic. Notwithstanding this issue, the high number of women in the diagnosed- and probably autistic groups provided greater power to examine the differences between them. Indeed, another important limitation of our research was its failure to recruit equivalent numbers of men, leading to a relatively small group of probably autistic men in Study 2. This restricted the conclusions we were able to draw about FPT, as it was not possible to ascertain whether the superiority of social functioning shown by probably autistic women compared to diagnosed autistic women was specific to females, as would be expected by FPT. Future studies should ensure sufficient recruitment of male participants. Saleh and Bista (2017) suggested that male recruitment rates for large-scale surveys can be improved if the questionnaire items are both short and concise. It is possible that the length and depth of our questionnaires, which also asked for personal and sensitive information relating to mental health, might have discouraged some men from taking part.

The present sample was also not fully representative of the UK’s general population. In Study 1, most participants were students, and while Study 2 did include more people in employment, they were still predominantly white British. A lack of representation from Black, Asian and Minority Ethnic (BAME) people is a common problem in autism research, and possibly a consequence of BAME autistic people not receiving appropriate support or diagnosis. This may reflect lack of awareness around autism, mistrust of health professionals, and increased stigma and shame associated with the diagnosis (NAS, 2014). Future studies should remove barriers that prevent BAME autistic people from participating in research to better understand autism in their communities (Kandeh et al., 2018). Additionally, future research could adopt a more nuanced approach to identity (e.g., with gender), and acknowledge the more complex intersectional identities of those within the autism community. This is an important consideration given that high rates of ‘gender variance’ have been found in autistic people (Cooper et al., 2018). Finally, it would be beneficial to confirm ASC diagnoses reported by participants who score below 32 on the AQ, rather than exclude them from the analyses, as this might help to identify the behavioural characteristics that lead to early ASC diagnosis despite less obvious autistic traits.

## Conclusions and Recommendations

In conclusion, our demonstration of an empathy and social functioning advantage among probably autistic women relative to diagnosed autistic women is in line with FPT. Additionally, our findings highlight the difficulties experienced by many autistic women in gaining timely ASC diagnosis. Based on the present findings, we make the following recommendations for clinicians. First, ASC should not be ruled out during psychiatric assessments because of seemingly typical interpersonal skills and social functioning, and full developmental histories should be taken that consider the patient’s experiences of masking autistic traits. Second, women presenting multiple times to clinicians with psychiatric difficulties should routinely be screened for autism, particularly those presenting with symptoms of BPD. Future research should also examine differences between diagnosed and probably autistic individuals in externalising behaviours, such as RRBIs, as these could be another important factor in identification (Dworzynski et al., 2012). It is crucial that diagnostic procedures for autism continue to be improved, particularly for women (Elder et al., 2017). Our findings showed clear impairments of social functioning in undiagnosed but probably autistic women relative to non-autistic women, suggesting they may be struggling considerably in everyday life without adequate support. A major concern for these women is their heightened risk of mental health difficulties (Cassidy et al., 2018). It is hoped that by putting into place the suggested recommendations, earlier diagnoses of ASC can be made, leading to more positive outcomes for many women.
